# Stochastic kinetics reveal imperative role of anisotropic interfacial tension to determine morphology and evolution of nucleated droplets in nematogenic films

**DOI:** 10.1038/srep40059

**Published:** 2017-01-05

**Authors:** Amit Kumar Bhattacharjee

**Affiliations:** 1 Centre for Condensed Matter Theory, Department of Physics, Indian Institute of Science, Bangalore 560064, India

## Abstract

For isotropic fluids, classical nucleation theory predicts the nucleation rate, barrier height and critical droplet size by ac- counting for the competition between bulk energy and interfacial tension. The nucleation process in liquid crystals is less understood. We numerically investigate nucleation in monolayered nematogenic films using a mesoscopic framework, in par- ticular, we study the morphology and kinetic pathway in spontaneous formation and growth of droplets of the stable phase in the metastable background. The parameter *κ* that quantifies the anisotropic elastic energy plays a central role in determining the geometric structure of the droplets. Noncircular nematic droplets with homogeneous director orientation are nucleated in a background of supercooled isotropic phase for small *κ*. For large *κ*, noncircular droplets with integer topological charge, accompanied by a biaxial ring at the outer surface, are nucleated. The isotropic droplet shape in a superheated nematic background is found to depend on *κ* in a similar way. Identical growth laws are found in the two cases, although an unusual two-stage mechanism is observed in the nucleation of isotropic droplets. Temporal distributions of successive events indi- cate the relevance of long-ranged elasticity-mediated interactions within the isotropic domains. Implications for a theoretical description of nucleation in anisotropic fluids are discussed.

A fluid exhibiting a first order phase transition can transit from an unstable to a stable phase through spinodal decomposition and coarsening, where irregular domains of the stable phase emerge spontaneously and combine to minimize the surface energy. In contrast, transformations from a metastable state occur via nucleation and growth in which droplets of the stable phase are formed in the metastable state and these droplets grow and coalesce to increase the fraction of the stable phase in the system. A classic example of this phenomenon is supercooled water freezing into ice via nucleation and growth^1^. Nucleation in solid solutions is followed by Ostwald ripening^2^, while metallic alloys and bulk metallic glasses conventionally display dendritic growth due to anisotropic surface effects^3^.

Many fundamental problems in surface interfacial science are concerned with the morphology of the nucleated phase, its growth rate, the first passage time as well as the kinetic route to equilibrium. Questions about droplet morphology are especially pertinent in studies of nematogenic fluids, where the anisotropy associated with the tensorial structure of the order parameter is one of the important factors in the description of the nucleation process^4^,^5^. The microstructure of the nucleus is determined by a nontrivial interplay of competing energies: (i) the anisotropic elastic energy associated with deformations of the tensorial order in the bulk, (ii) the anisotropic interfacial tension related to the director anchoring at the interface between the two phases, and (iii) any external forcing that may be present, e.g. equilibrium thermal fluctuations. Thus, aspherical shape of droplets, complex growth law etc. are to be expected and the nucleation rate may itself lack a precise definition^6^.

Recently, liquid crystalline phases have found a multitude of applications in nanoscience^7^. Droplet shapes play a crucial role in ink-jet technology^8^, switching and bistable devices^9^, photovoltaics as well as in bio-sensor applications with living liquid crystals^10^. Early experiments found evidence for aspherical spindle-shaped droplets called tactoids^11^. Such nuclei were later obtained in theoretical studies assuming homogeneous director distribution inside the droplet^12^,^13^,^14^. Progress was hindered for several decades because experimental characterization of early-stage supercritical droplets was not possible. Recently, long carbon nanotubes have been used in optical microscopy to characterize nematic tactoids^15^.

Computer simulations have traditionally played an important role in the development of an understanding of the kinetics of nucleation and growth. Computer simulations of nucleation processes have to address problems in defining the droplets unambiguously and in developing algorithms to sample rare events. Monte Carlo (MC) studies of hard spherocylinders have been performed, where ellipsoidal clusters with homogeneous director orientation are nucleated^4^,^16^. More recently, spherical nanodroplets with a radial hedgehog defect, accompanied by a Saturn-ring at the core and bipolar pole-centered boojum defects with uniform field structure have been reported^17^. It is worth mentioning that kinetic pathways in MC simulations can be misleading, as the algorithm samples the Gibbs distribution in equilibrium without obeying the natural dynamics of the system. Slower growth following a diffusive kinetics are reported in molecular dynamics (MD) simulation and experiments^18^,^19^. Recent studies have examined the morphology of freely suspended aspherical nanodroplets^20^. Although MD provides a comparatively well-defined temporal evolution than MC, nematic ordering is often best discussed using coarse-grained methods for which a top-down approach works very well^20^ due to the scale invariance of the dynamical equations, allowing its applicability from astrophysical scales, *e.g.* the Kibble-Zurek mechanism^21^,^22^, down to nanoscales. We use this approach in our work.

Nematic order is described by a symmetric, traceless tensor **Q**, which in component form reads^23^ *Q*_*αβ*_ = [*S*(3*n*_*α*_*n*_*β*_ − *δ*_*αβ*_) + *B*_2_(*l*_*α*_*l*_*β*_ − *m*_*α*_*m*_*β*_)]/2, where (*α, β*) ≡ (*x, y, z*) denote the Cartesian directions in a local frame of reference with 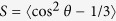 and 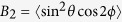 the scalar degree of uniaxial and biaxial order, respectively, (*θ, ϕ*) the polar and azimuthal angles and averaging is done over a sufficient large coarse-graining volume. [**n**, **l**, **m**] denote the director, codirector and secondary director forming an orthonormal triad. The Ginzburg-Landau-de Gennes (GLdG) free energy consists of a homogeneous bulk term and an elastic term representing the free-energy cost of distortions due to inhomogeneity, namely 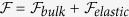, where


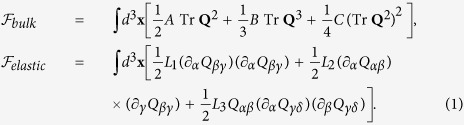



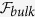
 is displayed in Fig. 1(A) that exhibits an asymmetric well landscape characterizing the weakly first order nature of the isotropic-nematic phase transition. The phase diagram in Fig. 1(B) is derived from 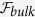, where the temperature dependence is contained in the parameter *A* = *A*_0_(1 − *T*/T ^*^) and the parameter *B* depends on the size disparity^24^. Minimizing 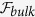 with respect to *S* yields the equilibrium value


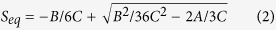


with the clearing point value *S*_*c*_ = −2*B*/9*C*.

The first two terms of 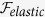 correspond to isotropic and anisotropic elasticity with the final term being a higher order contribution. The elastic constants *L*_1_, *L*_2_, *L*_3_ are obtained from experimental measures of Frank-Oseen splay (*K*_1_), twist (*K*_2_) and bend (*K*_3_) elastic constants via the relation^25^,


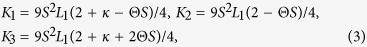


where *κ* = *L*_2_/*L*_1_ and Θ = *L*_3_/*L*_1_ (*L*_1_ > 0). Third order terms can be neglected (Θ = 0) leading to degenerate splay and bend with twist either large or small depending on the sign of *κ*^23^,^26^. Thus the GLdG theory loses its validity if bend and splay constants are very different. The *one elastic constant* approximation is often considered for analytic convenience, where *K*_1_ = *K*_2_ = *K*_3_ corresponds to *κ* = 0. However, experimental measures of elastic constants in units of 10^−7^ *dyn* and GLdG coefficients in units of *Jcm*^−3^ for (a) 5CB at 25 °C are *K*_1_ = 6.4, *K*_2_ = 3, *K*_3_ = 10, *B* = 7.2, *C* = 8.8 and (b) MBBA at 25 °C are *K*_1_ = 6, *K*_2_ = 4, *K*_3_ = 7.5, *B* = 2.66, *C* = 2.76^27^. Θ = 0 gives for 5CB, *L*_1_ = 0.649, *κ* = 40.667 and for MBBA, *L*_1_ = 8.6534, *κ* = 1.2. This explains why the *one elastic approximation* is inappropriate in a description of certain nematogenic materials.

Using this free energy, the geometric structure of monolayered droplets has been studied analytically in the past two decades, either making several simplifying assumptions^28^,^29^,^30^, or through an exact computation^31^. Going beyond the Frank-Oseen description of the elastic energy^32^ and without enforcing any phenomenological Rapini-Papoular (RP) surface energy term^33^, noncircular nematic droplets with integer topological charge have been found to grow ballistically^28^ in a deterministic (no thermal noise) calculation.

Homogeneous nucleation kinetics can not be studied in the deterministic GLdG framework because droplets of the stable phase cannot spontaneously nucleate in a metastable medium in the absence of thermal fluctuations. Near the transition point, droplet growth is governed by capillary forces rather than the small free energy difference or volume driving force, where fluctuations play a crucial role^34^. To understand how fluctuations influence the dynamics and microstructural evolution, one needs (i) the theoretical formulation of a stochastic GLdG description of the dynamics and (ii) a numerical prescription to integrate the stochastic equation for the orientation tensor^35^ paying special attention to the structure of the noise and satisfying the fluctuation-dissipation theorem (FDT). The first question was addressed by Stratonovich^35^ by writing an overdamped Langevin equation in model-A relaxational dynamics that excludes coupling to any external hydrodynamic flow as^36^,^37^


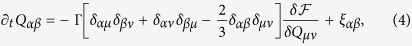


where the coefficient of rotational diffusion Γ controls the relaxation rate and the symmetric traceless tensorial random force ***ξ*** satisfies the property, 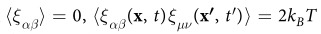 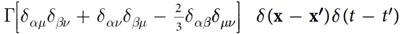 to ensure FDT and thus Gibbs distribution at equilibrium^38^. *k*_*B*_, *T* and brackets denote the Boltzmann constant, equilibrium temperature and average over the probability distribution of ***ξ***. The first term in 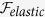 leads to *L*_1_∂^2^**Q** in equation (4), indicating an isotropic diffusion of **Q**. The second term in 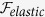 leads to 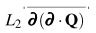 in the evolution equation, resulting in an orientation dependent **Q**-diffusion that leads to two diffusion constants in the nematic phase. The anisotropy is controlled by the parameter *κ* defined above. An efficient method for numerical integration of this equation was developed in a recent work of the author^37^ that motivated the present study.

Classical nucleation theory (CNT) estimates the critical size of a droplet, the barrier height and the nucleation rate using the assumption that nucleation proceeds via the formation and expansion of spherical droplets^39^,^40^. The excess free energy of a droplet is obtained as 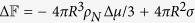, where *R* is the droplet radius, *ρ*_*N*_ is the density of the nucleated phase, 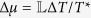 is the chemical potential difference with 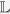 being the emitted latent heat due to a change in temperature Δ*T* and *σ* is the interfacial surface tension. Maximizing 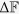 with respect to *R* yields *R*_*c*_ = 2*σ*/*ρ*_*N*_|Δ*μ*| and the barrier height 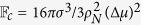. The nucleation rate is defined as 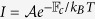 where 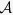 is a kinetic prefactor often hard to measure in experiments, making the rate calculation a formidable problem.

For droplets formed in a nematogenic material, due to the inherent anisotropy in the field variables, the free energy takes the form


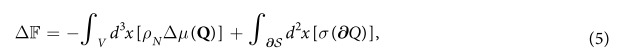


where *V* and 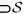 respectively denote the transformed volume and the enclosing surface. The complexity that renders an analytical insight difficult lies in the nontrivial coupling between principal values and principal axes of the **Q**-tensor. For an ellipsoidal droplet with homogeneous director distribution, analytic expressions can be derived from the above equation^16^,^41^ without considering a RP-term. However, for a noncircular droplet with an embedded defect, singular volume and surface integrals restrict the applicability of an analytic approach. The interfacial surface tension is thermodynamically defined as the excess surface energy per unit area. The first and second terms in 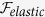 contribute to the isotropic and anisotropic parts of the surface energy, respectively. The excess anisotropic surface energy is controlled by the parameter *κ* defined earlier, that differentiates between strong and weak anchoring of the director at the interface.

Nucleation and growth are often characterized by the Johnson-Mehl-Avrami-Kolmogorov (JMAK) equation^42^,^43^,^44^ 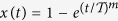, where *x(t*) is the volume fraction of the nucleated phase, *m* depends on the shape of the droplet and 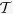 is a constant related to the growth velocity *v*. For isolated spherical droplets with number density *n*, simple analysis shows that 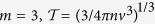. However, if we consider expanding ellipsoidal droplets where the long and the short axes increase self-similarly, then the parameters turn out to be 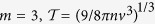. Higher exponents and fractional exponents are also seen in experiments and conventionally calculated through a plot of


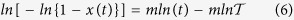


*versus ln(t*). While the exponent *m* is dictated by the dimensionality of the droplet, a departure from the predicted value suggests the inapplicability of simple theory and breakdown of CNT. In our results, droplet represents a quasi two-dimensional “raft”-like geometry formed in monolayered film.

## Results

We first discuss the tensorial microstructure and evolution of thermally generated nematic droplets in a supercooled isotropic phase. This is done for varying anisotropic surface energy and the results are compared with the predictions of classical theories of nucleation to test their applicability. We probe the role of long range elasticity mediated interaction on the distribution of the first passage time between successive events. We also consider the nucleation of isotropic droplets in a superheated nematic phase. The numerical values of the parameters used in our simulations are tabulated in Table 1.

### Nucleation in supercooled isotropic phase

Our central findings are summarized in terms of the droplet morphology, evolution of the **Q**-tensor and the free energy, growth law and temporal distribution of nucleation events. Figure 2 shows the supercritical droplet structure at the post-nucleation stage in terms of the uniaxial order *S* and the director distribution **n**, the biaxial order *B*_2_ and codirector distribution **l**, as well as the Schlieren texture for different values of *κ* chosen to ensure the positivity of the Frank elastic constants. The nucleated droplet in panel (B) is circular in the *one elastic constant* approximation (*κ* = 0) while the droplet in the weak anchoring limit (small *κ*) shown in panels (A, C) is noncircular. As indicated by the orientation of **n**, homeotropic anchoring at the interface is preferred for *κ* = −1, corresponding to *K*_2_ = 2*K*_1_ (defined in eq. (3)), where the uniform director inside the droplet orients perpendicular to the long axis. For negative values of *κ*, its magnitude cannot be arbitrarily large as an unphysical correlation length is numerically unavoidable for the analytical lower bound *κ* > −6^45^. On the other hand, planar anchoring is favoured for *κ* = 1 corresponding to *K*_2_ = 2*K*_1_/3, where the director orients parallel to the long axis. For a flat interface, the total energy is lowered for planar or homeotropic director anchoring for *K*_2_ being smaller or larger than *K*_1_. This result is often termed as the *de Gennes ansatz*^45^. Though this ansatz does not hold for curved interfaces (shown in the Supplementary Information), our results agree reasonably well with it. This result is also in agreement with deterministic GLdG calculations for bubbles created by hand^31^ and MC, MD simulations^4^,^20^. This result, however, contradicts those of ref. 28, where encapsulated integer-charged defects are reported inside an artificially constructed droplet for *κ* in the range (−4/7, 4/3) [parameter *K* in this study is related to *κ* by *κ* = 2*K*/(1 − *K*)]. Uniformly white textured domains in panels (D–F) are indicative of the homogeneous director distribution in panels (A–C). Finally, panels (G–I) illustrate that *B*_2_ has a small value (the order is uniaxial without any codirector or secondary director ordering) except for *κ* = −1. Biaxial fluctuations are visible in the isotropic film as 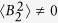 due to the presence of stochastic forcing.

This picture, however, changes dramatically in the strong anchoring limit (*κ* ≫ 0) as evident in the panels (J–L), where the microstructure at *κ* = 18, corresponding to *K*_2_ = *K*_1_/10, is depicted. Nonuniform director orientation inside the noncircular droplet corresponds to four-brush texturing that represents a hyperbolic hedgehog defect. The topological charge of −1 is quantified through a Volterra process^23^. This reveals that there exists a threshold value of *κ* ≫ 0, for which the surface anisotropy is large enough to distort the field structure inside the droplet to encapsulate a defect. While the generation and growth of a supercritical nucleus depends on the competition between bulk and surface contributions, with the latter increasing with *κ*, the shape and director configuration inside the nematic region strongly depend on the surface interfacial anisotropy. The codirector **l** and the secondary director **m** (not shown) also have a singular structure with *B*_2_ reaching a maximum on a noncircular ring embedded in the outer region of the droplet. This is consistent with the understanding that a planar interface exhibits local biaxiality for large *κ*^46^. When approximating the droplets to be circular, the critical droplet size can be estimated in terms of the parameters in the GLdG free energy. As mentioned in the caption of Fig. 2, unreasonable values of *R*_*c*_ are obtained as the droplets become more noncircular with a nonuniform director arrangement. However, no analytic formula for *R*_*c*_ can be obtained within a stochastic GLdG theory.

Next we address the various stages of the kinetics. Panels (A–D) of Fig. 3 illustrate the pre, post, intermediate and late stage structure of *S* and **n** for different *κ* and with large *L*_1_, implying droplets with a large surface energy. Increasing the barrier height results in a prolonged pre-nucleation stage and fewer supercritical droplets emerge in the post-nucleation period. Droplets grow self-similarly, coalesce at the intermediate stage and span the system at the late stage without forming any defect-antidefect pair. However, for smaller surface energy and *κ* ≤ 0, half-integer defects with two-brush textures emerge due to the coalescence of droplets that resembles a reduced uniaxial order within the defect core (see Supplementary Animation S1). The ordering kinetics proceeds via the annihilation of defects, thus reducing the total free energy of the film. For *κ* = 1, structures similar to boojum defects emerge at opposite poles of the droplet, where *S* has saturated to the equilibrium value without displaying any half-integer defects. For *κ* = 18, the nematic region gradually encroaches the isotropic domain with *S* saturating relatively quickly as compared to the integer defect annihilation kinetics. The four-brush texturing persists even at a very late stage without generating any two-brush texturing (see Supplementary Animation S2).

To understand the role of *κ* in the kinetics, the growth and decay of average uniaxial and biaxial ordering for small *L*_1_ are depicted in panel (E). The sigmoidal profile of 〈*S*〉 in the upper panel with higher intermediate slope inbetween two smaller slopes at early and late stages of the kinetics is a typical characteristic of nucleation followed by a growth process. For *κ* = −1, *t* < 10^3^ is identified as the pre-nucleation stage where subcritical nuclei shrink to zero, while *t* > 10^3^ denote the emergence of the supercritical nucleus and growth by agglomeration. Finally, the saturation of 〈*S*〉 for *t* > 3 × 10^3^ corresponds to the defect annealing process. As anticipated, the nucleation time is prolonged for increasing *κ*, resulting from increased surface energy and hence a higher barrier height. Thus the number of droplets decreases for higher surface anisotropy. The fraction of the stable phase, *x(t*) (0 < *x* < 1) and the function *Y* = *ln*[−*ln*{1 − *x(t*)}] are computed from the profile of 〈*S*〉 and fits to the JMAK equation (6) are displayed in the upper panel of (G). The intermediate slope indicated by the dashed grey lines with scaling exponent *m* > 2 indicates a breakdown of the simple theory and inapplicability of a CNT description. 〈*B*_2_〉 in the lower panel of (E) decreases in a step fashion as the nematic phase is approached. A nonzero biaxiality at equilibrium conveys a departure from a purely uniaxial nematic film, with the magnitude of 〈*B*_2_〉 decreasing with increasing *κ*. 〈*B*_2_〉 attains an intermediate maximum before a step decrease for *κ* ≫ 0. This is related to the coalescence of the biaxial rings shown in Fig. 2(L).

The total (free) energy of the film and the contributions from bulk and elastic energies are displayed in the panel (F). 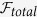 decreases monotonically with time. 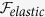 is smaller than 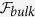 by about an order of magnitude. The elastic energy slowly increases and exhibits an overshoot before decreasing to attain the equilibrium value. The overshoot is maximized for *κ* = −1, arising from the coalescence of homeotropically anchored nematic droplets leading to a maximum in elastic energy. The overshoot gradually decreases with increasing *κ*, as less elastic energy is needed in combining planar anchored droplets.

The growth of the first nucleated cluster 〈*N*_*c*_〉 and the average cluster size at the post-nucleation stage before coalescence are displayed in the middle panel of (G). The growth law follows a polynomial form 〈*N*_*c*_〉 = *at*^2^ + *bt* + *c*, where *a, b, c* are fit parameters. As 〈*N*_*c*_〉 scales as the square of the characteristic length *L*, the growth law for a tagged cluster is predicted to be


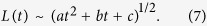


Evolution of the length scale for a tagged cluster, along with the average cluster size, is plotted in the lower panel of (G). In a brief period of the post-nucleation stage, the *at*^2^ term in eq. (7) can be neglected to obtain a diffusive, thermally limited regime where curvature elasticity and capillary forces play a more significant role than the free energy difference or the volume driving forces. Furthermore, the Laplace pressure is large due to a small radius of curvature and the surface interfacial tension, as well as the noncircular morphology of the droplet, induce local shear effects^26^,^34^,^47^,^48^. A crossover to a ballistic volume driven growth regime at a later stage, marked with a grey vertical line in the middle panel, where the *bt* term in eq. (7) can be neglected, corresponds to a propagating interface front before droplet coalescence. The late stage ballistic growth in deterministic spinodal kinetics in confined circular films has been addressed earlier with a crystal growth equation supplemented to the deterministic GLdG framework^34^. Experiments in confined geometry, however, find diffusive dynamics at long times, which is incorporated in the deterministic GLdG formalism along with the equation for latent heat at the interface. As the heated interfacial temperature becomes comparable to that of the nematic bulk, growth reaches a diffusive steady state with an equal rate of generation and diffusion of latent heat^6^,^19^. However, when the film is not confined, the latent heat effects are unimportant due to faster expulsion of heat from the droplet surface, leading to a long time ballistic growth.

To evaluate the validity of the CNT, we compute the nucleation rate as a function of the barrier height as sketched in Fig. 4. A significant departure from a decaying exponential signals a breakdown of the CNT. The CNT deals with the rate of phase change and growth of the supercritical cluster without accounting for fission and coalescence. Moreover, the occurrence of exponential dependency is expected in the Becker-Döring limit, *i.e.* near the coexistence line and for steady state rates. While deformation of the tensorial field due to high elastic anisotropy results in the formation of noncircular droplets, the theory can be applicable in the *κ* → 0 limit where director deformation is negligible and a circular shape is retained. In the weak anchoring limit (small *κ*), the CNT can still be applied if a noncircular shape is incorporated in the standard theory^16^ and the kinetic prefactor can be obtained from experimental results. However the CNT has to be supplemented to accommodate singularity in **n** in order to make it applicable in the strong anchoring limit (*κ* ≫ 0).

Finally to investigate the role of the isotropic medium on the temporal distribution of nucleation events, we study the spatiotemporal correlation between the first passage times of two consecutive nucleation events. Normalized histograms shown in Fig. 5 are sharply peaked for *κ* < 0 and the peak broadens for increasing *κ*. Also the distributions are correlated in time for *κ* < 0, although long-ranged elastic interactions are not present in the supercooled isotropic film. The reason for this correlation is not clear. The correlation disappears as *κ* is increased. Recall that in the isotropic phase, the correlation length is close to few grid spacings, so that events separated by more than that are unambiguously recognized as nucleation events. In the inset of panel (C), the spatial proximity of two such occurrences are shown. These events are temporally uncorrelated in spite of their spatial proximity. For *κ* = 18 both distributions coincide, indicating no memory of consecutive events.

### Nucleation in the superheated nematic phase

The isotropic droplet morphology, evolution of the **Q**-tensor, growth kinetics and temporal distribution of nucleation events have also been examined for the case where thermal fluctuations nucleate droplets of the isotropic phase in a superheated nematic film. Within feasible computational effort, nucleation of isotropic droplets can be obtained only for *κ* ≤ 6. Figure 6 displays the structure of supercritical droplets at the post-nucleation stage in terms of *S* and **n** for large *L*_1_ and different values of *κ*. Noncircular droplets nucleate for *κ* ≠ 0, while in panel (B) the droplet shape remains nearly circular for *κ* = 0 (*one elastic constant* approximation). The director distribution is randomized inside the droplet, indicating isotropy with no observable biaxiality. Unlike colloidal inclusion in a nematic medium^49^ or in nematic shells^50^, homeotropic anchoring at the interface by forming defects outside the droplet is not preferred.

**Figure 1:**
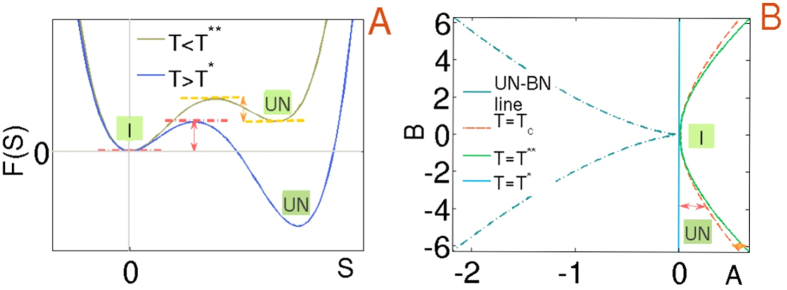
(**A**) Schematic illustration of the free energy with scalar order and (**B**) corresponding phase diagram with stable and metastable states. [I] and [UN] denote the isotropic and uniaxial nematic minima. Second order uniaxial-biaxial [UN-BN] line is also shown and the barrier height is marked in red (orange) for supercooling (superheating), with the spinodal temperatures^31^,^63^ denoted by T^*^, T^**^. Recall that T^*^, T^**^ and the clearing temperature *T*_*c*_ correspond to *A* = 0, *B*^2^/24*C* and *B*^2^/27*C* respectively^64^. For example in 5CB, T^*^ = 34.2 °C, T^**^ = 34.47 °C and *T*_*c*_ = 34.44 °C.

**Figure 2:**
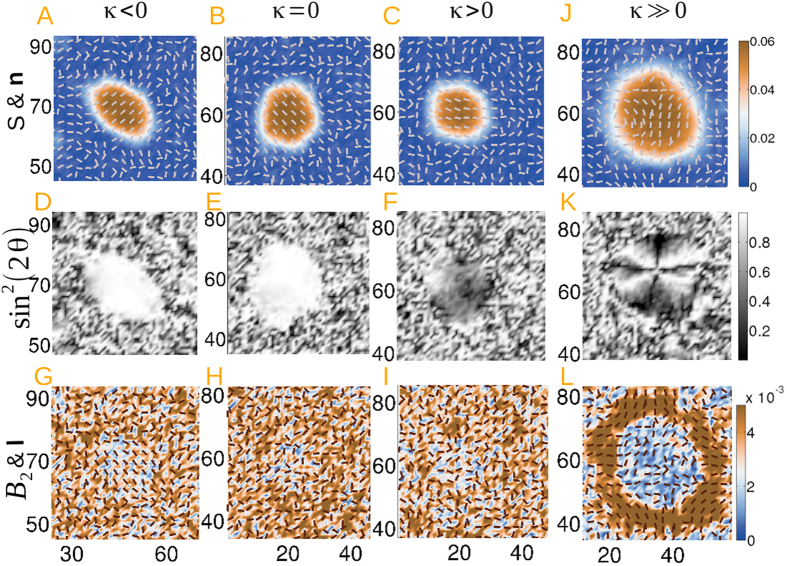
Nematic droplet structure in terms of the uniaxial order parameter and director orientation for *κ* = −1 at *t* = 4031*τ* (panel A), *κ* = 0 at *t* = 4623*τ* (panel B), *κ* = 1 at *t* = 6083*τ* (panel C) and *κ* = 18 at *t* = 8099*τ* (panel J) in the post-nucleation stage of the kinetics. Panels (D–F, K) display the corresponding Schlieren texture which is proportional to sin^2^(2*θ*) and panels (G–I, L) depict the degree of biaxiality and the codirector orientation. The critical radius for *L*_1_ = 0.01 and *κ* = (−1, 0, 1, 3, 6, 18) turns out to be *R*_*c*_ = (7.72, 9.35, 11.05, 11.8, 4.4, 0.49). Scalar field values are rendered in false colour.

**Figure 3:**
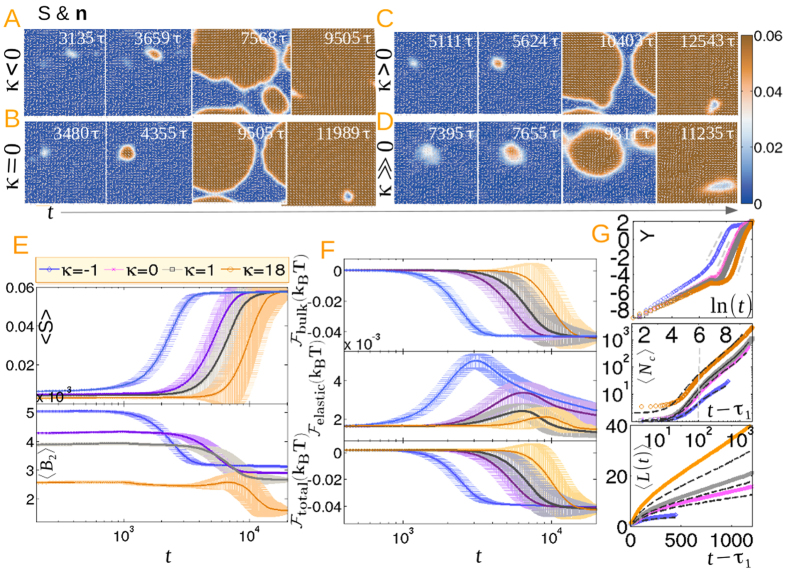
Panel (A–D): Evolution of the scalar uniaxial order and director structure at pre, post, intermediate and late stages of the kinetics for higher surface energy and different *κ* (See Supplementary Animations S1 and S2). Panel (E) displays the average uniaxial order 〈*S*〉 and biaxial order 〈*B*_2_〉 while panel (F) shows the bulk, elastic and total free energy of the film. Plots of the JMAK eq. (6) are displayed in the upper panel (G) with exponents *m* = (3.145, 3.425, 3.465, 3.98) for *κ* = (−1, 0, 1, 18) in ascending order. Finally, evolution of the number of points in a tagged cluster (coloured symbols) as well as the average cluster size (black dotted lines) for different *κ* are shown in the middle panel (G), while the lower panel (G) displays the evolution of the length scale obtained from the middle panel (G). 800 independent realizations are sampled to obtain the graphics in panel (E–G).

**Figure 4:**
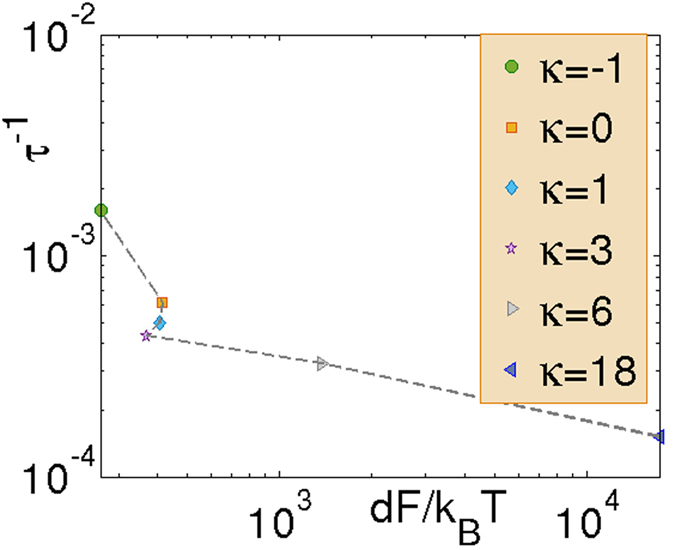
Nucleation rate as a function of barrier height for various *κ*. 400 realizations for each *κ* are sampled to obtain the graph.

To characterize the evolution process, panels (A–D) of Fig. 7 portray the pre, post, intermediate and late stage structure of *S* and **n** at a higher surface energy. Subcritical droplets form and collapse in the pre-nucleation stage while a supercritical droplet nucleates and expands self-similarly in the post-nucleation period. Droplet coalescence converts the film into a fluctuating isotropic state at the late stage of the kinetics. In a shallow quench where the surface energy and the barrier height is reduced, many small droplets are formed and they coalesce with each other. At a late stage, uniform regions of nematic order are squeezed and removed from the isotropic film. Rather surprisingly, 〈*S*〉 in panel (E) depicts of an unusual two-step decay process, while 〈*B*_2_〉 displays two minima. We interpret this observation in the following way (see Supplementary Animation S3). Quenching a uniform nematic medium to metastability at a higher temperature induces fluctuations that decrease the scalar order parameter. The plateau in 〈*S*〉 corresponds to its “quasi-equilibrium” value in the superheated metastable state. For smaller surface energy and a reduced barrier height, the typical size of regions of fluctuation-induced melting is comparable to the critical droplet size. Therefore, the subcritical droplets do not shrink to zero but persist for sufficient amount of time at the pre-nucleation stage, until fluctuations induce the formation of a supercritical droplet. Several other mechanisms for the slowing down of the decay of 〈*S*〉 may be present, for instance (i) fluctuation induced broadening of the zero curvature value of the superheating line in Fig. 1, (ii) higher Laplace pressure arising from small droplets, (iii) curvature elasticity and capillary force effects. Local heating due to the emission of latent heat at the droplet surface can be ignored, while such effects become important at higher droplet radius in confined geometry^19^,^47^. As the minimum in 〈*S*〉 corresponds to the maximum in 〈*B*_2_〉, two minima separated by a plateau occurs in the lower panel of (E). The post-nucleation droplet growth due to agglomeration is displayed in the upper panel of (G) which is characterized by the JMAK equation, with the slope sketched in grey dashed lines. Scaling exponents *m* > 2 indicate to a breakdown of the CNT description.

Further support of the two-stage growth process is provided in the evolution of energy as highlighted in panel (F). 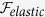 slowly decreases after exhibiting two overshoots, with the prominent one at a late stage before saturating to the equilibrium value. The overshoot corresponds to a maximum in the elastic energy during droplet coalescence. Both bulk and total free energies display a plateau where growth remains temporally frozen. As is evident, the plateau increases with increasing *κ*, indicating that more surface energy slows down the formation of supercritical nuclei. These effects are more evident when higher surface energy is considered (see Supplementary Animations S3 and S4) where due to increased barrier height, the critical radius is large compared to the fluctuation-induced melted droplets and subcritical droplets disappear quickly from the film.

To quantify the growth process, we explore the evolution of a tagged cluster and the average cluster size 〈*N*_*c*_〉. Middle and lower panels of (G) display them for *κ* = 1 and 6. For *κ* < 1, it was impossible to keep track of single clusters due to very small correlation length. Evolution of the average cluster length, shown in the inset of the middle panel, is found to follow the tagged cluster dynamics. The growth law is observed to obey eq. (7) with a change of an early diffusive to a late stage ballistic growth before coalescence.

**Figure 5:**
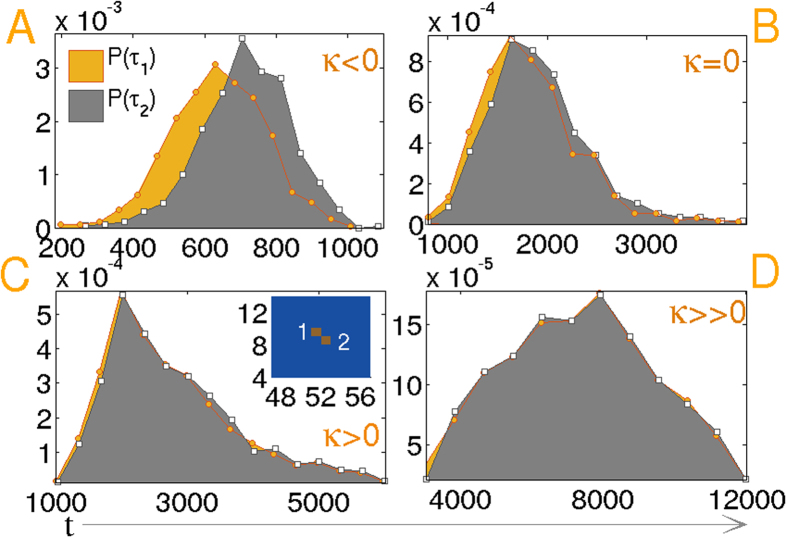
(**A**–**D**) Normalized probability distribution *P(τ*_1_) of the first nucleation event ‘1’ is displayed along with the probability distribution *P(τ*_2_) of the consecutive event ‘2’ for increasing *κ* = (−1, 0, 1, 18). The spatial proximity of two events is shown in the inset of panel (C). 800 temporal points are sampled to obtain the histograms.

**Figure 6:**
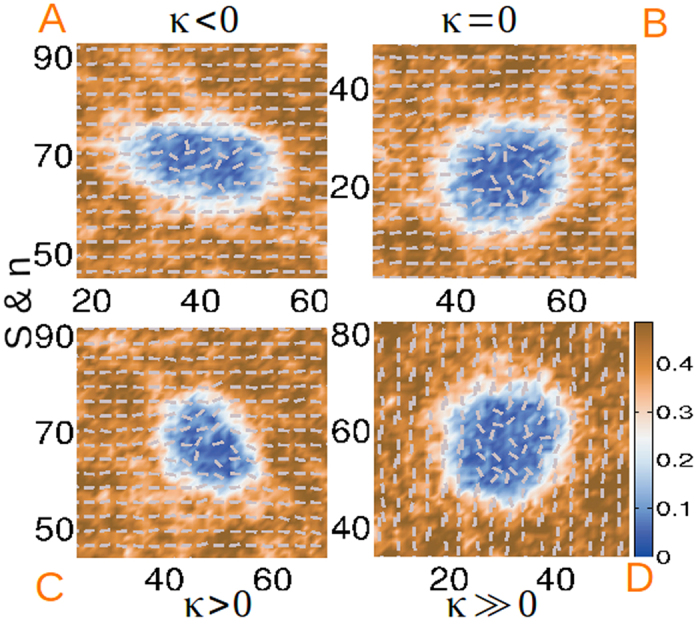
Isotropic droplet structure in terms of the uniaxial order parameter and director arrangement in the post-nucleation stage of the kinetics, for (**A**) *κ* = −1 at time *t* = 22051*τ*, (**B**) *κ* = 0 at time *t* = 52211*τ*, (**C**) *κ* = 1 at time *t* = 21903*τ* and (**D**) *κ* = 6 at time *t* = 147839*τ*. Scalar field values are rendered in false colours.

**Figure 7:**
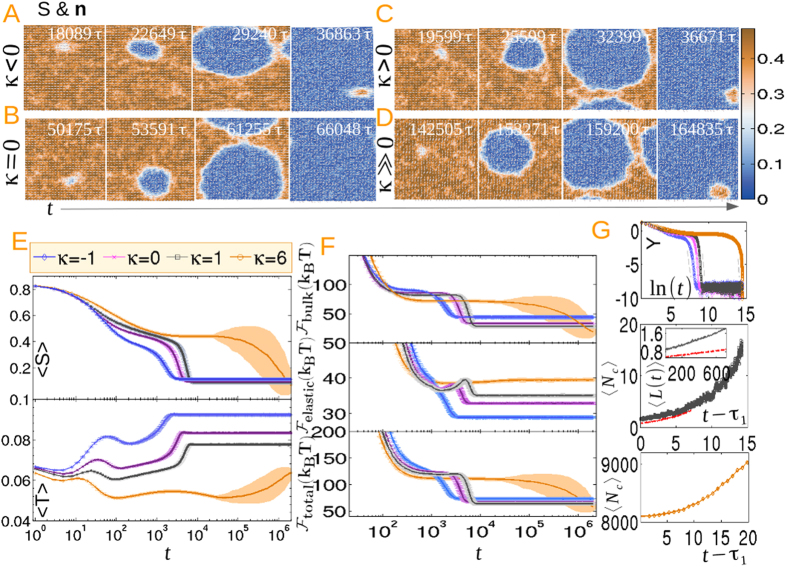
Panel (A–D): Evolution of uniaxial order and director orientation at different stages for higher surface energy and different values of *κ* (see Supplementary Animation S3 and S4). Panel (E) displays the evolution of the average uniaxial and biaxial order while panel (F) shows bulk, elastic and total energy of the superheated film. The upper Panel in (G) presents fits to the JMAK equation with exponents *m* = (2.862, 2.952, 3.197, 4) for *κ* = (−1, 0, 1, 6) in ascending order. Middle and lower panels in (G) depict evolution of 〈*N*_*c*_〉 for *κ* = 1 and 6. The x-axis corresponds to (*t* − *τ*_1_) × 10^2^ for *κ* = 1 (middle panel) and (*t* − *τ*_1_) × 10^3^ for *κ* = 6 (lower panel). Growth of tagged cluster size (black line) and average cluster size (red dotted line) are shown in the inset of the middle panel in (G). Total 100 independent realizations are sampled to procure the graphics.

**Figure 8:**
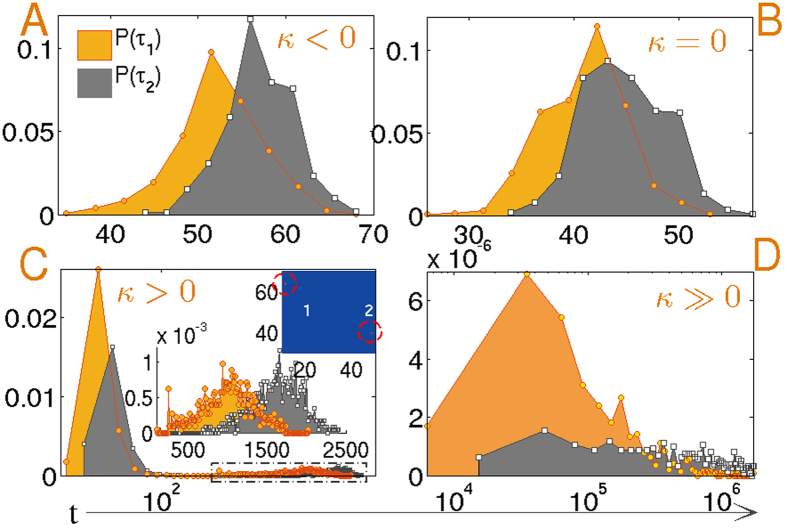
(**A**–**D**) Normalized probability distributions [*P(τ*_1_), *P(τ*_2_)] of first and subsequent nucleation events at times *τ*_1_ and *τ*_2_ for ascending values of *κ* = (−1, 0, 1, 6). The second peak of the bimodal distribution is amplified in the inset of panel (C) where the spatial separation of events ‘1’ and ‘2’ is also portrayed. Histograms are made with 800 independent points for panel (A–C) while 500 points were sampled to obtain panel (D).

To examine the role of spatial long-ranged interactions in the first passage times of consecutive events, we study the spatiotemporal correlation between the events. Figure 8 sketches the normalized histograms for different *κ*. The distribution is sharply peaked within a small temporal domain for *κ* = −1, and the span of the distribution increases by two orders of magnitude with significant broadening as *κ* is increased. The first and second events are always correlated due to the long range elastic interaction in the nematic film. The bimodality exhibited by the distributions for *κ* = 1 is surprising. As seen in the amplified plot in the inset of panel (C), the second peaks are also correlated. The reason for bimodality can be physically understood as the limit in which the size of the regions of fluctuation induced melting becomes less than the critical droplet size. Thus the first peak in the histogram in panel (C) results from the initial formation of subcritical droplets that disappear in time. However, supercritical droplets nucleate at a later stage displayed in the upper inset, with two consecutive events marked as ‘1’ and ‘2’ that are spatially distant but temporally correlated. This should be compared with the inset of Fig. 5(C), where although events ‘1’ and ‘2’ are spatially proximate, they are temporally independent. Due to higher surface energy for *κ* ≫ 0, a single droplet nucleates and the events in panel (D) are monomodal, but still correlated with a much wider temporal distribution compared to that in the weak anchoring limit, shown in panel (A–C).

## Discussion

We have performed an extensive study of homogeneous nucleation kinetics in a freely suspended monolayer of metastable liquid crystalline film using stochastic nematodyamics. In the case of a supercooled film in the metastable isotropic phase, we have shown that the presence of a large surface interfacial anisotropy quantified by a large value of the parameter *κ* leads to the appearance of a noncircular droplet of the nematic phase with an encapsulated hyperbolic hedgehog defect and a biaxial interfacial ring as seen in 5CB microdroplets^19^. Noncircular droplets exhibit homogeneous orientation of the director field for smaller values of *κ*. The growth of the nuclei at small volumes is found to exhibit a polynomial dependence on time. The regime of applicability of classical nucleation theories in the small *κ* limit is determined. Also, successive nucleation events are found to be uncorrelated even if they are spatially proximate, due to the absence of long ranged elastic interactions in an isotropic film. On the other hand, a two step growth process is observed in isotropic droplet nucleation in a superheated nematic film. In this case, spatially distant nucleation events are temporally correlated due to the long ranged elastic interactions in the nematic film. These findings are consistent with available results in three spatial dimensions^51^, but are markedly different from the results of studies of confined films where coverslips affect the director component in the third direction^28^.

The kinetic pathway of ordering from an unstable isotropic phase to a stable nematic phase through spinodal decomposition and coarsening in a deterministic GLdG framework has been extensively studied^31^,^51^,^52^,^53^,^54^,^55^ in the past. In this case, the development of diffusive domains and late-stage defect pair kinetics (Porod law regime) take place at a much faster time scale compared to nucleation kinetics. When a nematic film is heated to a temperature above the superheating line, disordered isotropic domain coarsening leads to a stable isotropic state^19^. A comparison of existing results for late-stage domain growth in these cases with those obtained from the stochastic GLdG framework considered here is outside the scope of the present study. Also, electrokinetic^56^ and flexoelectric effects^57^ as well as coupling of the orientation tensor to a hydrodynamic flow field^58^ for a thermal system can be considered in the future. Other choices for describing biaxial order^59^ may be explored in future investigations. Experimental verification of the results reported here would be welcome, although avoiding heterogeneous nucleation when sampling rare events in a narrow temperature window is a challenging task.

## Methods

### Numerical integration of stochastic GLdG equation

A two dimensional monolayer of nematogenic material is considered, where orientation in three Cartesian directions is retained, but spatial variations are restricted to a plain. The **Q**-tensor equation is solved on a regular square lattice with periodic boundary condition to neglect confinement effects. A direct numerical integration is forbidden as using similarity transformation, any symmetric traceless tensor cannot be diagonalized at every grid point. By utilizing a property that the tensor can be expanded in a basis of five 3 × 3 matrices **T**^60^, a legitimate way is to project the equations as **Q** = **∑**_*i*_*a*_*i*_**T**_*i*_ and ***ξ*** = **∑**_*i*_*ζ*_*i*_**T**_*i*_ (*i* = 1, …, 5), so as to contain the dynamics in the basis coefficients *a*_*i*_(**x**, *t*) and *ζ*_*i*_(**x**, *t*)^37^. Major advantage is gained in constructing the symmetrized detraced noise ***ξ*** with five *ζ*_*i*_, that corresponds to zero mean unit variance independent Gaussian white noise processes. This thus validates discrete FDT spectrum in all Fourier modes and we obtain reasonable agreement in static and dynamic correlations of **Q** with analytic formula both in isotropic and nematic phase^37^. Eq. (4) in the basis coefficients takes the form


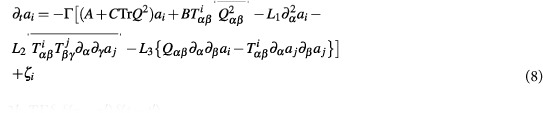


where 〈*ζ*_*i*_(**x**, *t*)*ζ*_*j*_(**x**′, *t*′)〉 = 2*k*_*B*_*T* Γ*δ*_*ij*_*δ*(**x** − **x**′)*δ(t* − *t*′).

Laplacian and mixed derivatives are spatially discretized as 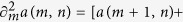

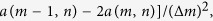
 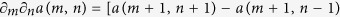 − 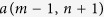, 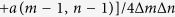 where *m*, **n** denote Cartesian indices. We adopt second order accurate stochastic method of lines (SMOL) integrator for explicit temporal update^61^. SMOL semi-discretization scheme develops on discretizing spatial part of partial differential equations to yield ordinary time-dependent equations, which are integrated on unstructured grid maintaining accuracy, stability and computational overload.

The distortion free energy, length and time are resolved by transforming the deterministic part of eq. (4) in non-dimensionalized form to obtain 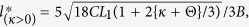 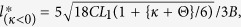 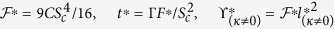, where 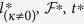 and 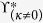 are non-dimensional length, bulk energy, time and surface energy. Dimensional quantities for example, correlation length and relaxation time can be computed as 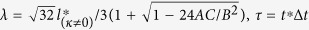. To avoid numerical artifact, 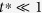 and *λ* ≫ Δ*x* are strictly maintained. Also 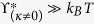 is ensured to avoid the medium to attain the stable phase in one computational step.

### Cluster labelling procedure

To sample nucleation clusters, we record results on every computational step within a time window within which the cluster eventuates. We apply Hoshen Kopelman (HK76) algorithm^62^ to label connected clusters on the grid which are above (below) certain threshold. To identify nematic nuclei, we choose threshold value at 70% of *S*_*eq*_ and implement periodicity in both directions to overcome double counting of connected clusters through periodic boundaries. In case of isotropic nucleation, the algorithm performs reversely and we choose the threshold value at 30% of *S*_*eq*_. The algorithm particularly finds usefulness in counting the total number of grid points pertaining to a tagged cluster that temporally amplifies as the cluster swells. Thus a length scale can be simply extracted to quantify growth law, without computing the length scale from direct correlation functions^31^ that also captures Porod law scaling of defect annealing kinetics after droplet coalescence.

## Additional Information

**How to cite this article**: Bhattacharjee, A. K. Stochastic kinetics reveal imperative role of anisotropic interfacial tension to determine morphology and evolution of nucleated droplets in nematogenic films. *Sci. Rep.* **7**, 40059; doi: 10.1038/srep40059 (2017).

**Publisher's note:** Springer Nature remains neutral with regard to jurisdictional claims in published maps and institutional affiliations.

## Supplementary Material

Supplementary Material

Supplementary Animation S1

Supplementary Animation S2

Supplementary Animation S3

Supplementary Animation S4

## Figures and Tables

**Table 1:** 

Fig.	Γ(*Poise*^−1^)	*A(Jcm*^−3^)	*B(Jcm*^−3^)	*C(Jcm*^−3^)	*L*_1_(10^−7^ *dyn*)	*κ*	*λ(μm*)
(2, 3A–3D)	1	10^−3^	−0.5	2.67	(0.025, 0.012, 0.012, 0.01)	(−1, 0, 1, 18)	(3.38, 2.56, 3.31, 8.44)
(3E–3G, 4, 5)	1	10^−3^	−0.5	2.67	0.01	(−1, 0, 1, 18)	(2.14, 2.34, 3.02, 8.44)
(6, 7A–7D)	1.25 × 10^−2^	0.38019	−4.0	1.67	(1.5, 0.895, 0.66, 0.4)	(−1, 0, 1, 6)	(3.95, 3.35, 3.71, 5)
(7E–7G, 8)	1.25 × 10^−2^	0.38019	−4.0	1.67	0.4	(−1, 0, 1, 6)	(2.04, 2.24, 2.89, 5)
							
**Fig.**	Υ^*^		**Fig.**		Υ^*^	***k***_***B***_***T**(**J***)	t^*^
(2, 3A–3D)	(1.80, 1.04, 1.73, 11.3) × 10^−5^		(3E–3G, 4, 5)		(2.89, 8.66, 14.4, 113) × 10^−6^	2.0807 × 10^−7^	2.6 × 10^−3^
(6, 7A–7D)	(1.77, 1.27, 1.56, 2.83) × 10^−1^		(7E–7G, 8)		(4.72, 5.67, 9.44, 28.3) × 10^−2^	6 × 10^−3^	3.33 × 10^−3^

Values of parameters used to obtain the plots shown in Figs 2, 3, 4, 5, 6, 7 and 8, where a box of size *L*_*x*_ = *L*_*y*_ = 96 *μm* with grid spacing Δ*x* = Δ*y* = 1 *μm* and time step Δ*t* = 1 *μs* are considered. For 5CB, the data correspond to a temperature of 34.27 °C in Figs (2, 3, 4 and 5) and 34.46 °C in Figs (6, 7 and 8). Definition of the parameters are given in the Methods section.
