# Supplementary material for: Stochastic kinetics reveal imperative role of anisotropic interfacial tension to determine morphology and evolution of nucleated droplets in nematogenic films
Source: Sci Rep. 2017 Jan 5;7:40059. doi: 10.1038/srep40059 (PMC5213422; doi:10.1038/srep40059)
Supplement: Supplementary Material [file srep40059-s1.pdf]

Supplementary Information : Stochastic kinetics reveal imperative role of anisotropic interfacial tension to determine morphology and evolution of nucleated droplets in nematogenic films

Amit Kumar Bhattacharjee[1]

[1] *Centre for Condensed Matter Theory, Department of Physics, Indian Institute of Science, Bangalore 560064, India*

(Dated: November 25, 2016)

Breakdown of *de Gennes ansatz* on curved surface interface

In a principal frame, the diagonal components of $\mathbf{Q}$ are written as, $Q_{xx} = -(S+T)/2, Q_{yy} = -(S-T)/2, Q_{zz} = 0$. The matrix can be transformed to a fixed frame of reference by rotation with pitch angle θ and yaw angle ϕ to obtain, $Q_{xx} = -\big\{(S+T)\cos^2\phi\cos^2\theta + (-S+T)sin^2\phi cos^2\theta + 2Ssin^2\theta\big\}/2, Q_{xy} = -Tsin(2\phi)cos\theta/2, Q_{xz} = \big\{3S + Tcos(2\phi)\big\}sin(2\theta)/4, Q_{yy} = \big\{-S + Tcos(2\phi)\big\}/2, Q_{yz} = Tsin(2\phi)sin\theta/2, Q_{zz} = \big[2S - 2Tcos(2\phi) + Tcos\big\{2(\phi-\theta)\big\} + 6Scos(2\theta) + Tcos\big\{2(\phi+\theta)\big\}\big]/8$. Inhomogeneities of S, T, θ and ϕ are encoded in $\mathcal{F}_{elastic}$, whose minimization for different κ decides stable director anchoring at surface interface.

In absence of thermal fluctuations ($\boldsymbol{\partial}\theta, \boldsymbol{\partial}\phi = 0$) for a planar I-N interface along z-direction where the director is confined to a plain ($\phi = 0$), the anisotropic elastic energy takes the form

$$\mathcal{F}_{anelastic} = \kappa\big[sin^2(2\theta)(3\partial_z S + \partial_z T)^2 + \big\{1 + 3cos(2\theta)\times \partial_z S - 2sin^2\theta\partial_z T\big\}^2\big]/32. \quad (1)$$

Thus, free energy is lowered for homeotropic anchoring ($\theta = 0$) for $\kappa < 0$ and planar anchoring ($\theta = \pi/2$) for $\kappa > 0$, in par with de Gennes argument [1]. However for a curved interface,

$$\mathcal{F}^{(\theta=0)}_{anelastic} = \kappa\big[(\partial_z S)^2 + \big\{\partial_y S/2 + sin(2\phi)\partial_x T/2 + (1/2 - cos\phi)\partial_y T\big\}^2 + \big\{\partial_x S + cos(2\phi)\partial_x T + sin(2\phi)\partial_y T\big\}^2/4\big]/2,$$
$$\mathcal{F}^{(\theta=\pi/2)}_{anelastic} = \kappa\big[(\partial_x S)^2 + \big\{-\partial_z S + sin(2\phi)\partial_y T - cos(2\phi)\partial_z T\big\}^2/4 + \big\{-\partial_y S + (-1/2 + cos\phi)\partial_y T + sin(2\phi)\times \partial_z T/2\big\}^2\big]/2, \quad (2)$$

which can be further reduced in a quasi two-dimensional geometry by taking $\partial_z S, \partial_z T = 0$. Depending on the sign of κ and according to the competing values of the gradients in S, T and ϕ, the film decides the favoured anchoring. Accounting to thermal fluctuations ($\boldsymbol{\partial}\theta, \boldsymbol{\partial}\phi \neq 0$), director anchoring at the droplet surface interface is not intuitive.

Time-lapse animations & Captions

Animation S1 : *Circular nematic droplets for $\kappa = 0$ and lower surface energy.*

Description: The animation sequentially portrays evolution of (a) S & $\mathbf{n}$, (b) T & $\mathbf{l}$ and (c) Schlieren texture in *one elastic approximation* and for small values of elastic constant L_1. Nucleation of circular nematic bubbles with uniform director field is observed, that amplify in size to coalesce with other droplets. Note that many droplets are formed as in shallow quench, and droplet coalescence resulted into defects of half integer charge due to lower surface energy. For higher surface energy (or larger L_1), only few droplets are nucleated whose coalescence does not generate defects (not shown). Almost no notable change in T and $\mathbf{l}$ fields is seen in the process. Finally, schlieren texture depicts the uniformity of director field within the droplets.

Animation S2 : *Noncircular nematic droplets with encapsulated hyperbolic hendgehog defects for $\kappa \gg 0$ and higher surface energy.*

Description: The animation sequentially portrays evolution of (a) S & $\mathbf{n}$, (b) T & $\mathbf{l}$ and (c) Schlieren texture in strong anchoring limit and for higher L_1. Nucleation of noncircular nematic bubbles with encapsulated defect is observed, that amplify in size to coalesce with other droplets. The formation of biaxial ring at the droplet interface with hyperbolic hedgehog defect structure is also observed in T and $\mathbf{l}$ fields. Finally, schlieren texture depict the 4-brush geometry, that persist at very late stage of the kinetics.

Animation S3 : *Double occurance of noncircular isotropic droplets for $\kappa = 1$ and lower surface energy.*

Description: The animation sequentially portrays evolution of (a) S & $\mathbf{n}$ and (b) Schlieren texture in weak anchoring limit and for lower L_1. Almost no notable

change in T and $\mathbf{l}$ field is seen and thus omitted from the animation. Nucleation of noncircular isotropic bubbles without any defect in the bulk nematic film is observed. Note the double occurance of droplets, resulting to bimodality in the probability distribution of nucleation events. Nucleated droplets in later stage amplify in size to coalesce with other droplets and span the system size to form isotropic phase. Schlieren textures depict the nonuniformity of the director field more transparently.

Animation S4 : *Noncircular isotropic droplets for* $\kappa = 6$ *and higher surface energy.*

Description: The animation sequentially portrays evolution of (a) S & $\mathbf{n}$ and (b) Schlieren texture in strong anchoring limit and for higher L_1. Nucleation of noncircular isotropic bubbles is observed that amplify in size to coalesce with other droplets. Schlieren textures support the nonuniformity of director field within the droplets and absence of defects in the nematic environment, as well as in the squeezing nematic domains at the late stage of the kinetics.

[1] P. G. de Gennes, Molecular Crystals and Liquid Crystals **12**, 193 (1971).
